# Abundance of *P*-glycoprotein and Breast Cancer Resistance Protein Measured by Targeted Proteomics in Human Epileptogenic Brain Tissue

**DOI:** 10.1021/acs.molpharmaceut.1c00083

**Published:** 2021-05-19

**Authors:** Aniv Mann Brukner, Sarah Billington, Mony Benifla, Tot Bui Nguyen, Hadas Han, Odeya Bennett, Tal Gilboa, Dana Blatch, Yakov Fellig, Olga Volkov, Jashvant D. Unadkat, Dana Ekstein, Sara Eyal

**Affiliations:** † Institute for Drug Research, School of Pharmacy, Faculty of Medicine, The Hebrew University of Jerusalem, Room 613, Ein Kerem, Jerusalem 91120, Israel; ‡ Department of Pharmaceutics, School of Pharmacy, University of Washington, Seattle, Washington 98195, United States; § Children’s Neurosurgery Department, Rambam Academic Hospital, Haifa 31999, Israel; ∥ Department of Pediatrics, Shaare Zedek Medical Center, Jerusalem 91031, Israel; ⊥ Neuropediatric Unit, Pediatrics Division, Hadassah-Hebrew University Medical Center, Jerusalem 91120, Israel; # The Faculty of Medicine, The Hebrew University of Jerusalem, Jerusalem 91120, Israel; ∇ Department of Neurology, Agnes Ginges Center for Human Neurogenetics, Hadassah Medical Organization, Jerusalem 91120, Israel; ○ Department of Pathology, Hadassah-Hebrew University Medical Center, Jerusalem 91120, Israel; ◆ Nuclear Medicine Institute, Sheba Medical Center, Tel Hashomer 52621, Israel

**Keywords:** antiseizure medications, antiepileptic drugs, epilepsy, targeted proteomics, *P*-glycoprotein, breast cancer resistance protein

## Abstract

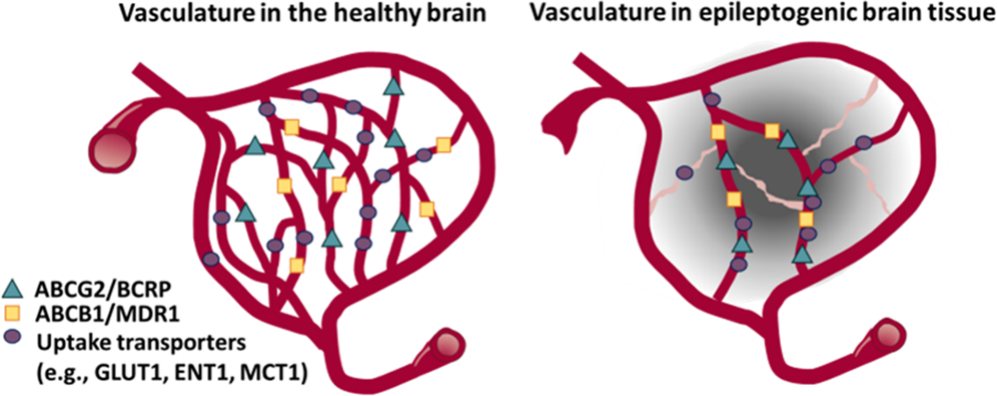

Our goal was to measure the absolute differential abundance of key drug transporters in human epileptogenic brain tissue and to compare them between patients and at various distances from the epileptogenic zone within the same patient. Transporter protein abundance was quantified in brain tissue homogenates from patients who underwent epilepsy surgery, using targeted proteomics, and correlations with clinical and tissue characteristics were assessed. Fourteen brain samples (including four epileptogenic hippocampal samples) were collected from nine patients. Among the quantifiable drug transporters, the abundance (median, range) ranked: breast cancer resistance protein (ABCG2/BCRP; 0.55, 0.01–3.26 pmol/g tissue) > *P*-glycoprotein (ABCB1/MDR1; 0.30, 0.02–1.15 pmol/g tissue) > equilibrative nucleoside transporter 1 (SLC29A1/ENT1; 0.06, 0.001–0.35 pmol/g tissue). The ABCB1/ABCG2 ratio (mean 0.27, range 0.08–0.47) was comparable with literature values from nonepileptogenic brain tissue (mean 0.5–0.8). Transporter abundance was lower in the hippocampi than in the less epileptogenic neocortex of the same patients. ABCG2/BCRP and ABCB1/MDR1 expression strongly correlated with that of glucose transporter 1 (SLC2A1/GLUT1) (*r* = 0.97, *p* < 0.001; *r* = 0.90, *p* < 0.01, respectively). Low transporter abundance was found in patients with overt vascular pathology, whereas the highest abundance was seen in a sample with normally appearing blood vessels. In conclusion, drug transporter abundance highly varies across patients and between epileptogenic and less epileptogenic brain tissue of the same patient. The strong correlation in abundance of ABCB1/MDR1, ABCG2/BCRP, and SLC2A1/GLUT1 suggests variation in the content of the functional vasculature within the tissue samples. The epileptogenic tissue can be depleted of key drug transport mechanisms, warranting consideration when selecting treatments for patients with drug-resistant epilepsy.

## Introduction

Drug-resistant epilepsy, defined as the failure of two or more appropriately chosen and used antiseizure medications (ASMs) to control seizures, is associated with increased risks of injuries, premature death, and reduced quality of life.^1,2^ A prevailing theory of drug resistance in epilepsy attributes the failure of ASMs to reach their targets to overexpression of efflux transporters, particularly *P*-glycoprotein (ABCB1/MDR1), in epileptogenic brain tissue.^2−9^ The expression of the other major blood–brain barrier (BBB) “gatekeeper”, the breast cancer resistance protein (ABCG2/BCRP), is unaffected by epilepsy.^10,11^ Additional efflux transporters, multidrug resistance-associated proteins (MRPs), have been shown to be upregulated in epileptogenic tissue, but their abundance in the human brain is much lower as compared to that of ABCB1/MDR1 and ABCG2/BCRP.^12−14^ Almost all of studies have comparatively and not absolutely quantified transporter protein expression in the epileptogenic focus within the human or rodent brain. In addition, although drug resistance might as well result from reduced uptake from the blood, this mechanism has only scarcely been explored in epilepsy.^13,15^

Quantitative targeted proteomics, using liquid-chromatography-tandem mass spectrometry (LC–MS/MS) is a promising approach for predicting tissue drug concentrations by extrapolating *in vitro* studies to *in vivo*.^12,16^ This method has been used before for quantifying transporter expression in brain tissue resected from epilepsy patients^13^ and provided important data as to their absolute expression on isolated blood vessels. However, that study did not involve samples from the epileptogenic focus but from as far away as possible from it.

Here, we used targeted proteomics to quantify, for the first time, whole-tissue abundance of key drug transporters in the epileptogenic zone of surgically resected brain tissue from patients with drug-resistant epilepsy. Our goal was to better understand mechanisms that govern drug delivery to the epileptic brain and estimate the variability between and within patients in transporter availability. Specifically, we were interested in addressing the following questions: (1) Is the ABCB1/ABCG2 abundance ratio higher than the values previously observed in the healthy human brain (as would be expected based on overexpression of ABCB1/MDR1 but not ABCG2/BCRP)? (2) Are drug transporters differently abundant in epileptogenic and less epileptogenic human tissue? We additionally correlated absolute transporter abundance to the clinical characteristics of the patients and the brain samples.

## Methods

### Patients

All patients 15 years old or older who underwent epilepsy surgeries at the Hadassah Hebrew University Medical Center between February 2, 2015 and August 18, 2016 and signed an informed consent prior to the surgery were included in the study. One patient was later excluded from the final analysis due to inadequate labeling of the resected tissue samples. Eight patients underwent resective neurosurgeries for drug-resistant epilepsy and an additional patient underwent surgical resection of an epileptogenic vascular lesion. Video-electroencephalography (EEG) monitoring, MRI scans, and a neuropsychological battery were part of their evaluation. Additionally, [^18^F]fluorodeoxyglucose (FDG)-positron emission tomography (PET)-computed tomography (CT) scans were conducted in eight patients, and images were analyzed visually before surgery.

The tissue to be resected during surgery was determined according to the customary clinical practice based on the combined data obtained from the various presurgical evaluations and in some cases, further intra- (eCog) or extraoperative (grid) invasive direct electrophysiological recording from exposed cortical areas (see “surgical procedure” in Table 1 for details). Finally, the precision in the location and the extent of resection were determined retrospectively based on the clinical outcome of the patient (“Engel” in Table 1). Of note, as widely accepted in clinical practice, when the epileptogenic area was believed to reside in the mesial temporal structures (i.e., the amygdala and the hippocampus), additional anterior temporal neocortical (NC) resection was carried out as part of the surgery (patients 1, 3, 5, and 8 in Table 1).

**Table 1:** Patient and Disease Characteristics

patient number	1	2	3	4	5	6	7	8	9
patient age at the time of surgery (year)	31	19.5	17	32	38	39	25	22	29
sex	female	male	female	male	female	male	male	female	male
medical history	pulmonary embolism in pregnancy	febrile seizures	none	left frontal calcified vascular malformation	none	schizophrenia-like disorder	preterm delivery, perinatal anoxia	depression	perinatal hypoxia, attention deficit hyperactivity disorder
epilepsy (yr)	30	5	16	17	37	17	14	19	12
medications taken in the month before surgery	carbamazepine, lamotrigine, levetiracetam atorvastatin, acetaminophen, phenylephrine	oxcarbazepine	carbamazepine	carbamazepine, phenobarbital, levetiracetam, valproic acid	levetiracetam, lamotrigine	phenobarbital, carbamazepine, topiramate, clotiapine	valproic acid, lamotrigine, levetiracetam, esomeprazole	phenobarbital, carbamazepine, levetiracetam, clonazepam	levetiracetam, valproic acid, clobazam
MRI	left mesio-temporal sclerosis	right cavernoma	right mesio-temporal sclerosis	left lesion, gliosis	right mesio-temporal sclerosis	left frontal cavernoma	left temporal-occipito-parietal ischemic lesion	left mesio-temporal sclerosis, temporal dysplasia	right dysplasia
[^18^F]FDG PET	global left temporal hypometabolism, more in medial regions	not performed	mild right temporal mesial hypometabolism	not performed	global right temporal hypometabolism, more in anterior and medial regions	left antcingulate hypometabolism near cavernoma	left posterior and mild anterior left temporal hypometabolism	diffuse hypometabolism in left temporal lobe spreading to parietal lobe	right temporal hypometabolism, slight global asymmetry
seizure frequency before surgery	1–2/day	0.5/month	1–4/month	1–2/week	3–6/month	2–4/month	1–3/day	2–3/week	1–2/week
surgical procedure	left anterior temporal lobectomy, AHC	right temporal lesionectomy, cortectomy, eCog	right anterior temporal lobectomy, AHC	left frontal lesionectomy, cortectomy, eCog	right anterior temporal lobectomy, AHC	left frontal cavernous angioma resection, eCog	left parieto-occipital- lesionectomy, grid	left anterior temporal lobectomy, AHC	right anterior temporal lobectomy, eCog
pathology	hippocampal sclerosis, ILAE type 1; neocortex: hyaline thickening of some blood vessel walls, neuropil loosening, lymphocyes and macrophages	meningioangiomatosis, calcific mass, aberrant vasculature	hippocampal sclerosis ILAE type 1; normal neocortex	residual calcified vascular malformation, scar-like gliosis in previous craniotomy site	hippocampal sclerosis, ILAE type 1; neocortex: mild gliosis, microgliosis	cavernous angioma	remote hypoxic/ischemic injury	hippocampal sclerosis ILAE type 1; neocortex: mild hyaline thickening of some blood vessel walls, neuropil loosening, lymphocytes and macrophages	thickening of blood vessel walls, neuropil loosening, few lymphocytes, hemosiderophages
Engel^a^, time from surgery (year)	IID (4.6)	IC (4.2)	IIC (4.2)	IVB (4.2)	IA (4.3)	IIIA (3.3)	IC (3.8)	IA (2.8)	IA (3.8)

a Class I: free of disabling seizures; class II: rare disabling seizures; class III: worthwhile improvement; and class IV: no worthwhile improvement. For subcategories of the Engel surgical outcome scale, see Engel.^19^ Neuropil: dense gray matter network of fine glial processes, neuronal processes, and fibrils;AHC: amygdalohippocampectomy; and ILAE: International League Against Epilepsy.

Clinical, demographic, and ancillary data were obtained from the neurologists (D.E., O.B., and T.G.) and the neurosurgeon (M.B.) involved in the cases and from the retrospective review of electronic medical records. Seizure frequency was categorized as high, intermediate, or low for daily, weekly, or monthly incidence, respectively.

The study was approved by the Hadassah Medical Center’s Institutional Review Board (Protocol #0322-13-HMO). Written informed consent was obtained from participants or their legal guardians prior to surgery.

### Tissue Collection and Handling

Most of the brain tissue resected during surgery was formalin-fixed for pathological evaluation (as part of standard clinical management) without any orientation markers and thus arbitrarily grossed and entirely embedded in paraffin blocks. The paraffin blocks were cut to 4 μm thick paraffin sections and stained with hematoxylin and eosin according to standard protocols. The sections were revisited by the pathologist (Y.F.) who was blinded to the other findings, after completion of the other analyses. The remaining small brain tissue samples were freshly placed in sterile tubes containing 15–30 mL of ice-cold sterile phosphate-buffered saline (PBS; Biological Industries, Beith Haemek, Israel). The tubes were kept on ice for a maximum of 25 min before weighing in preweighed tubes, snap-freezing in liquid nitrogen, and storage at −80 °C. Samples (0.182–2.014 g) were shipped to the University of Washington by a designated courier (World Courier, Stamford, CT, United States) using dry ice, which was refreshed daily.

### Lysate Preparation and Trypsin Digestion

Transporter abundance was measured in whole-cell lysates (not isolated endothelial cells due to low sample weights) based on a previously published protocol.^12^ Briefly, homogenates were prepared from whole-brain tissue samples in Dulbecco’s modified Eagle medium (DMEM)/F12 medium. The homogenates were washed twice using Dulbecco’s phosphate-buffered saline and then centrifuged.

Homogenates were incubated in lysis buffer (EB2: 2% sodium dodecyl sulfate, 1:1, protease inhibitor, and DNase I) overnight at 4 °C. Protein concentrations of the lysates were measured by the Pierce bicinchoninic acid (BCA) protein assay. Protein was reduced, denatured, alkylated, precipitated, and trypsin-digested over 16 h at 37 °C (enzyme:substrate ratio, 3.2:220 μg).^12,17^ The digests were combined with labeled internal standard (labeled surrogate peptides) and formic acid and centrifuged at 4000*g* for 5 min. The supernatant was transferred to an LC–MS/MS vial for analysis.

### Calibrator and Quality Control Samples

The surrogate peptides used for protein quantification were selected as we described before.^12^ For the creation of calibrator samples and quality control samples, 50 mM ammonium bicarbonate was spiked with unlabeled surrogate peptide standards, labeled internal standard peptides, and formic acid. In parallel to homogenate lysates, a 160 μg pool of the human liver protein (a biological quality control) was trypsin-digested and analyzed by LC–MS/MS, as detailed above.

### LC–MS/MS-Based Quantification of Transporter Protein Abundance in Tissue Lysates

The LC–MS/MS analysis was conducted using a Waters Xevo TQS tandem mass spectrometer coupled to an Acquity UPLC system (Waters Technologies, Milford, MA) operated in an electrospray positive ionization mode, under the same conditions and using the same gradients as we described before.^12^ In brief, the samples injected on to the Waters Acquity UPLC HSS T3 column (1.8 μm 100A; 100 × 2.1 mm) were equivalent to 21 μg (brain lysates) or 15 μg (liver) of protein. Mobile-phase composition was 0.1% formic acid in water (A) and 0.1% formic acid in acetonitrile (B). Gradient (0.3 mL/min) parameters were as follows: 0–3 min: 3% B; 3–10 min: 13% B; 10–20 min: 25% B; 20–24 min: 50% B, 24–24.1 min: 80% B; 24.1–25 min: 80% B; 25–25.1 min: 3% B; and 25.1–30 min: 3% B. Multiple-reaction monitoring analysis was used for detection. The mass spectrometry conditions were set at capillary 2 kV, offset source 60 V, and source temperature 350 °C.

LC–MS/MS data were processed using Waters MassLynx Software 4.1, as described previously.^12,18^ The calibration curve for the surrogate peptide was linear (*R*^2^ > 0.99) with a lower limit of quantification (LLOQ) of 0.35 fmol-on-column. The acceptable percentage error and the coefficient of variation was ±20% for quality control samples.^12^

Transporter protein abundance was calculated according to the following equation
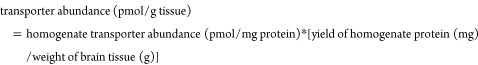
The performers of the proteomic analyses were blinded to the characteristics of the patients.

### PET Image Analysis

[^18^F]-activity was defined in images from diagnostic, nondynamic [^18^F]FDG-PET images, coregistered to pre- and postsurgical MR images. Image coregistration and manual drawing of spherical volumes of interest (VOIs) were conducted using Syngo.via VB10B (Siemens AG, Munich, Germany), guided by a nuclear medicine specialist (O.V.). The VOIs were drawn over the resected pathological areas, to correspond with the resected epileptogenic tissue and over the cerebellum. To reflect the clinical setting, values were not corrected for the partial volume. Because the volume of the [^18^F]FDG solution remaining in the syringe postinjection was unknown, we did not use absolute values. Instead, peak values (kBq/mL) of each VOI drawn over the resected regions were normalized by the mean of ipsilateral and contralateral cerebellar VOIs of the same study.

### Statistical Analysis

Statistical analysis was performed using the Spearman correlation, linear regression, and two-tailed Wilcoxon matched-pairs signed-rank test (Prism 6, GraphPad, La Jolla, CA). The significance level was set at *p* < 0.05.

## Results

### Patient and Disease Characteristics

Nine patients underwent neurosurgical resection of the assumed epileptogenic zone. Their demographic and clinical data are summarized in Table 1. At the time of surgery, their median age was 29 years (range, 17–39 years) and the median duration of epilepsy was 17 years (range, 5–37 years). Four patients underwent resection of the anterior temporal lobe, the amygdala, and the hippocampus for mesial temporal sclerosis (two samples/patient). The surgical procedure in the other patients was aimed at resection of the epileptogenic lesion with intraoperative electrocorticography or extraoperative invasive monitoring using subdural electrodes (one or two samples/patient).

At follow-up of more than 3 years in almost all cases, seven patients had favorable outcomes, with five of them being free of disabling seizures (Table 1).

### Transporter Abundance

**Figure 1:**
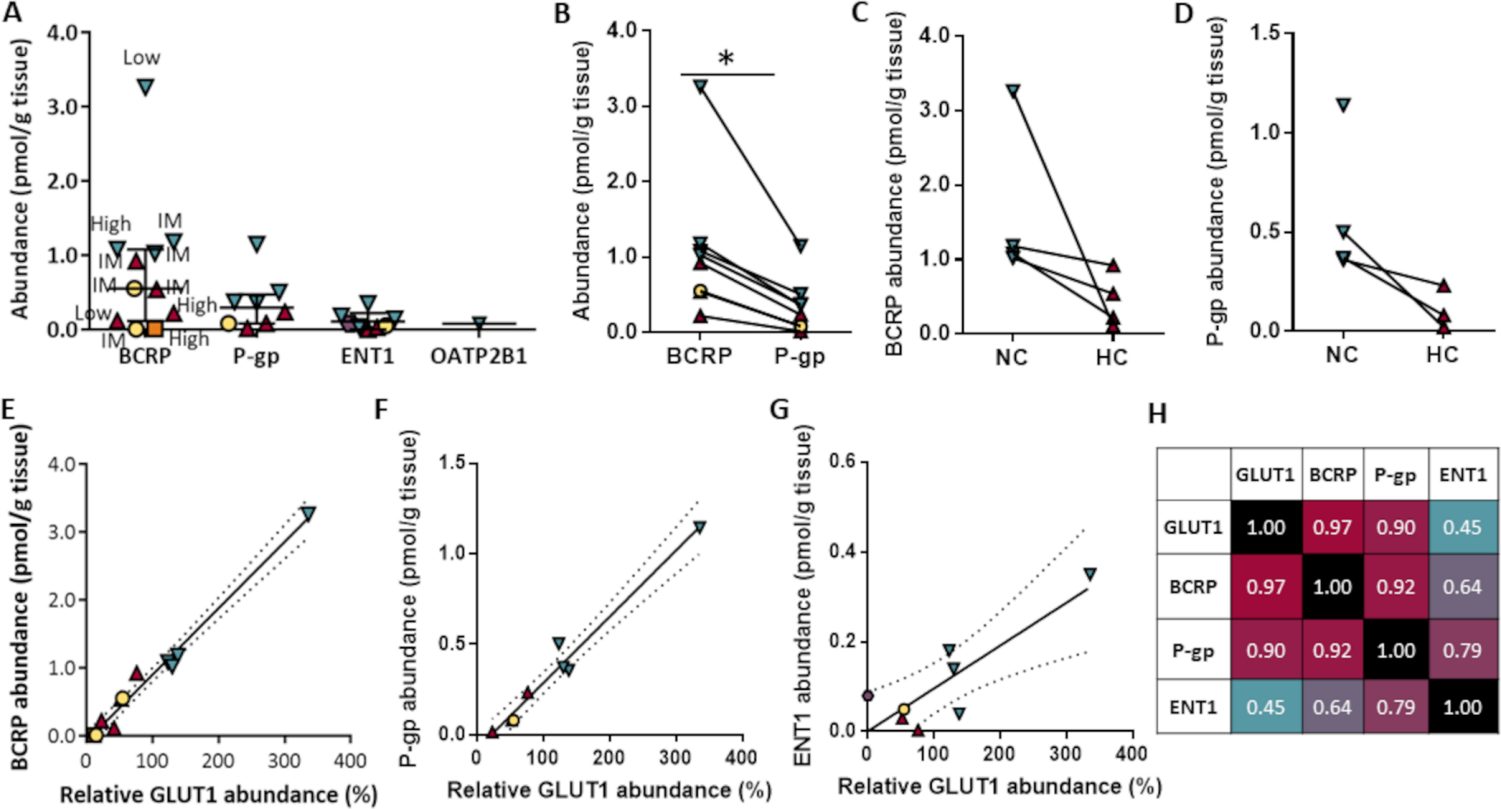
Transporter protein abundance in epileptogenic brain tissue (based on the data presented on Table 2). (A) Comparative levels of ABCG2/BCRP, ABCB1/MDR1 (*P*-gp), SLC29A1/ENT1, and SLC21A9/OATP2B1. Results are presented as median and interquartile range of 11, 8, and 8 samples in which ABCG2/BCRP, ABCB1/MDR1 (*P*-gp), and SLC29A1/ENT1 were quantifiable, respectively. SLC21A9/OATP2B1 was above the LLOQ in one sample. Triangles, hippocampal (HC) tissue from patients with hippocampal sclerosis; inverse triangles, temporal neocortex from the same patients; circles, two samples from a patient with right temporal dysplasia (no. 9); hexagon, temporo-occipital lobe tissue from a patient with meningioangiomatosis (no. 2); and square, occipito-temporal lobe tissue from a patient with an ischemic lesion (no. 7). Most patients continued antiseizure medications throughout the surgery, but one (no. 7) underwent drug withdrawal 4 days before the surgery, after implantation of the subdural electrodes. Also shown are relative seizure frequencies before surgery with regard to each sample. High, ≥1 seizure/day; IM, intermediate, <1 seizure day–>1 seizure week; and low, an average of <1 seizure/week. (B) Abundance of ABCG2/BCRP and ABCB1/MDR1 in individual tissue samples. *Statistically significant difference, *p* < 0.01, Wilcoxon matched-pairs signed-rank test. (C, D) ABCG2/BCRP (C) and ABCB1/MDR1 (D) abundance in hippocampal (HC; right) and adjacent neocortical (NC; left) tissue in four patients with hippocampal sclerosis. Each line connects data from individual patients. ABCB1/MDR1 was below the lower limit of quantification (LLOQ) in one hippocampal tissue sample. SLC29A1/ENT1 was below LLOQ in two hippocampal samples and is not shown. (E) GLUT1-BCRP correlation. (F) GLUT1-MDR1 correlation. (G). GLUT1-ENT1 correlation. The dashed lines in (E–G) represent the 95% confidence band. (H) Heatmap of protein–protein correlation coefficients (*r*; Spearman) across the four studied transporters.

Out of 15 transporters, ABCG2/BCRP, ABCB1/MDR1, equilibrative nucleoside transporter 1 (SLC29A1/ENT1), and organic anion transporting polypeptide 2B1 (SLC21A9/OATP2B1) were quantifiable (above LLOQ) in 11, 8, 8, and 1 samples, respectively. Median abundance of drug transporters ranked: ABCG2/BCRP (0.55 pmol/g tissue)> ABCB1/MDR1 (0.30 pmol/g tissue) > SLC29A1/ENT1 (0.06 pmol/g tissue) (Table 2 and Figure 1A). ABCG2/BCRP abundance was consistently higher as compared to that of ABCB1/MDR1 across all samples (*p* < 0.01; Figure 1B). The difference remained significant when the outlying neocortical tissue was excluded (Figure S1). The ratio of ABCB1/ABCG2 protein abundance (median 0.28, mean 0.27, range 0.08–0.47) was not higher than in nonepileptogenic human brain tissue (mean 0.5–0.8).^13,14^ Likewise, the ratio in four temporal lobe samples (outside the epileptogenic focus) was not higher than that reported by Shawahna et al. in isolated blood vessels^13^ (0.36 vs 0.65, respectively). The SLC2A1/GLUT1 expression highly varied across samples. The mean protein yield of the homogenates (4.6 mg protein/g tissue, Table 2) was lower by more than an order of magnitude than in homogenates from nonepileptic brains (82.0–87.2 mg protein/g tissue).^12^

**Table 2:** Tissue Characteristics and Transporter Abundance

				brain protein abundance (pmol/g tissue)				abundance (relative)^a^
patient no.	brain region	tissue weight (mg)	homogenate protein yield (mg protein/g tissue)	ABCG2/BCRP	ABCB1/MDR1	SLC29A1/ENT1	SLC21A9/OATP2B1	SLC2A1/GLUT1
1	anterior temporal lobe	437.1	3.9	1.08	0.50	0.18	<LLOQ	123.6
1	posterior hippocampus	227.0	4.7	0.22	0.02	<LLOQ	<LLOQ	23.2
2	temporo-occipital lobe	556.5	3.1	<LLOQ	<LLOQ	0.08	<LLOQ	
3	posterior temporal lobe	360.0	5.7	3.26	1.14	0.35	0.08	336.0
3	hippocampus	182.0	5.3	0.11	<LLOQ	<LLOQ	<LLOQ	42.1
4	frontal lobe	218.5	4.3	<LLOQ	<LLOQ	<LLOQ	<LLOQ	
5	posterior temporal lobe	269.8	6.0	1.18	0.36	0.04	<LLOQ	138.7
5	anterior hippocampus	222.3	6.0	0.92	0.23	0.001	<LLOQ	76.5
6	posterior frontal lobe	156.0	3.1	<LLOQ	<LLOQ	<LLOQ	<LLOQ	
7	occipital-temporal lobe	199.3	3.0	0.01	<LLOQ	<LLOQ	<LLOQ	12.2
8	posterior temporal lobe	302.3	5.5	1.02	0.37	0.14	<LLOQ	130.1
8	hippocampus	242.1	4.5	0.54	0.08	0.03	<LLOQ	52.4
9	anterior temporal lobe	244.7	5.3	0.55	0.08	0.05	<LLOQ	55.1
9	posterior temporal lobe	266.3	3.9	0.01	<LLOQ	<LLOQ	<LLOQ	15.1
median		266.3	4.6	0.55	0.30	0.06		55.1
mean		337.0	4.6	0.81	0.35	0.11		91.4

a Percent of mean SLC2A1/GLUT1 expression. ABCB1/MDR1, *P*-glycoprotein; ABCG2/BCRP, breast cancer resistance protein; LLOQ, lower limit of quantification; OATP, organic anion transporting polypeptide; SLC2A1/GLUT1, glucose transporter 1; and SLC29A1/ENT1, equilibrative nucleoside transporter.

ABCG2/BCRP and ABCB1/MDR1 expression was lower in the hippocampus compared with the adjacent neocortex in each patient with hippocampal sclerosis, but the numbers were too small for statistical comparisons (Figure 1C,D). Lower transporter expression appeared to be associated with high seizure frequency (Figure 1A). Transporter expression did not correlate with the duration of epilepsy (*p* > 0.05; not shown).

**Figure 2:**
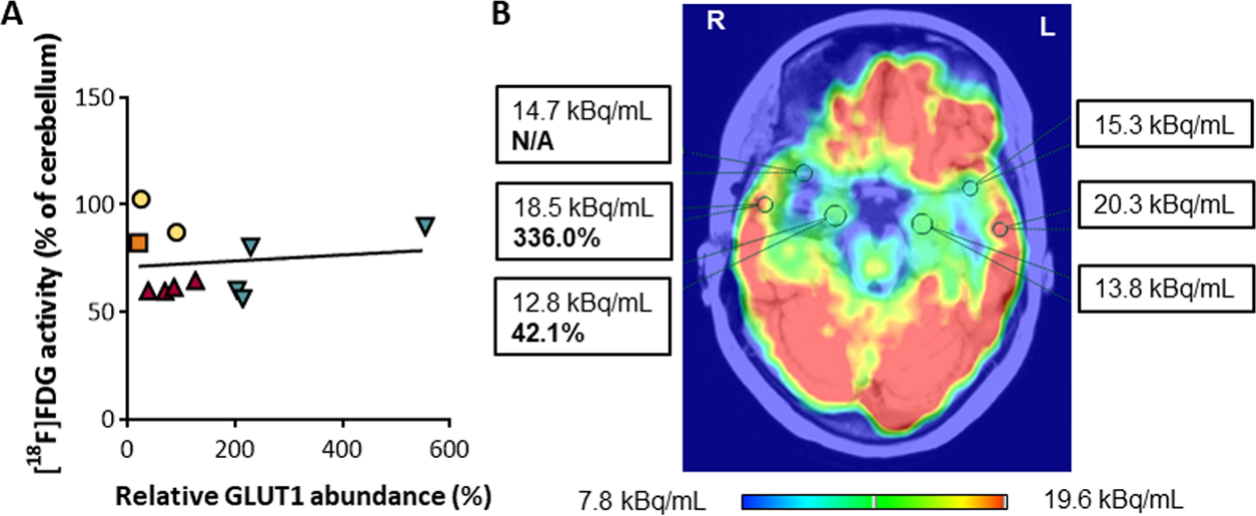
[^18^F]-activity in epileptogenic and reference brain tissue. (A) Correlation between relative SLC2A1/GLUT1 expression and [^18^F]FDG activity ([^18^F]FDG in resected tissue/cerebellar signal) across patients and samples. (B) Presurgical [^18^F]FDG-PET brain image of patient 3 (highest neocortical SLC2A1/GLUT1 expression) superimposed on postsurgical MRI, showing resection of the right anterior temporal lobe and of the right amygdala and hippocampus. Numerical values represent the [^18^F]FDG activity within respective VOIs and SLC2A1/GLUT1 abundance in tissue resected from the hippocampus and the posterior temporal lobe (bold). The [^18^F]FDG activity in the right hippocampus was lower than in the left hippocampus (93%), the right posterior temporal lobe (69%), and right anterior temporal lobe (87%). No asymmetry was observed in the cerebellum (not shown). In comparison, SLC2A1/GLUT1 abundance in the right hippocampus was 12.5% of the abundance in the right posterior temporal lobe.

SLC2A1/GLUT1 protein abundance was quantifiable in 12 samples from seven patients, but its absolute abundance could not be calibrated due to missing data. Hence, SLC2A1/GLUT1 data are presented as values relative to the mean of all patient samples in which it was quantifiable. Post-hoc analysis indicated that ABCG2/BCRP and ABCB1/MDR1 expression covaried and strongly correlated to that of SLC2A1/GLUT1 (*r* = 0.97, *p* < 0.001 for ABCG2/BCRP; *r* = 0.90, *p* < 0.01 for ABCB1/MDR1; Figure 1E,F,H). Modest correlation was observed between SLC29A1/ENT1 and SLC2A1/GLUT1 abundance (*r* = 0.45, *p* > 0.05; Figure 2G,H). The respective *R*^2^ values were 0.98, 0.97, and 0.72. The BCRP-GLUT1 and MDR1-GLUT1 correlations remained significant when the abovementioned outlier was excluded (*r* = 0.97, *p* < 0.001 and *r* = 0.86, *p* < 0.05, respectively; Figure S2).

The SLC2A1/GLUT1-efflux transporter correlation suggested that the three transporters are coexpressed at the BBB, and that normalization of efflux transporter abundance by that of SLC2A1/GLUT1 may control for tissue vascularization. Following the normalization, the ranks of ABCB1/MDR1 and ABCG2/BCRP abundance across samples changed only modestly (Figure S3), but the intersample variability (coefficient of variation) in transporter abundance was reduced (from 114 to 55% for ABCG2/BCRP and from 104 to 46% for ABCB1/MDR1).

### [^18^F]FDG-PET-CT Findings

Given that [^18^F]FDG radioactivity can reflect the SLC2A1/GLUT1 function^20,21^ and to assess the feasibility of estimating transporter abundance based on clinical [^18^F]FDG-PET-CT, we analyzed the PET images obtained for presurgical patient evaluation. [^18^F]FDG activity in resected brain tissue normalized to that of the cerebellum did not correlate to SLC2A1/GLUT1 abundance (*r* = −0.31, *p* > 0.05, Figure 2A). That is, the magnitude of reduction in SLC2A1/GLUT1 abundance did not necessarily translate into a parallel decrease in [^18^F]FDG activity, even within the same patient (Figure 2B).

### Blood Vessel Characteristics

**Figure 3:**
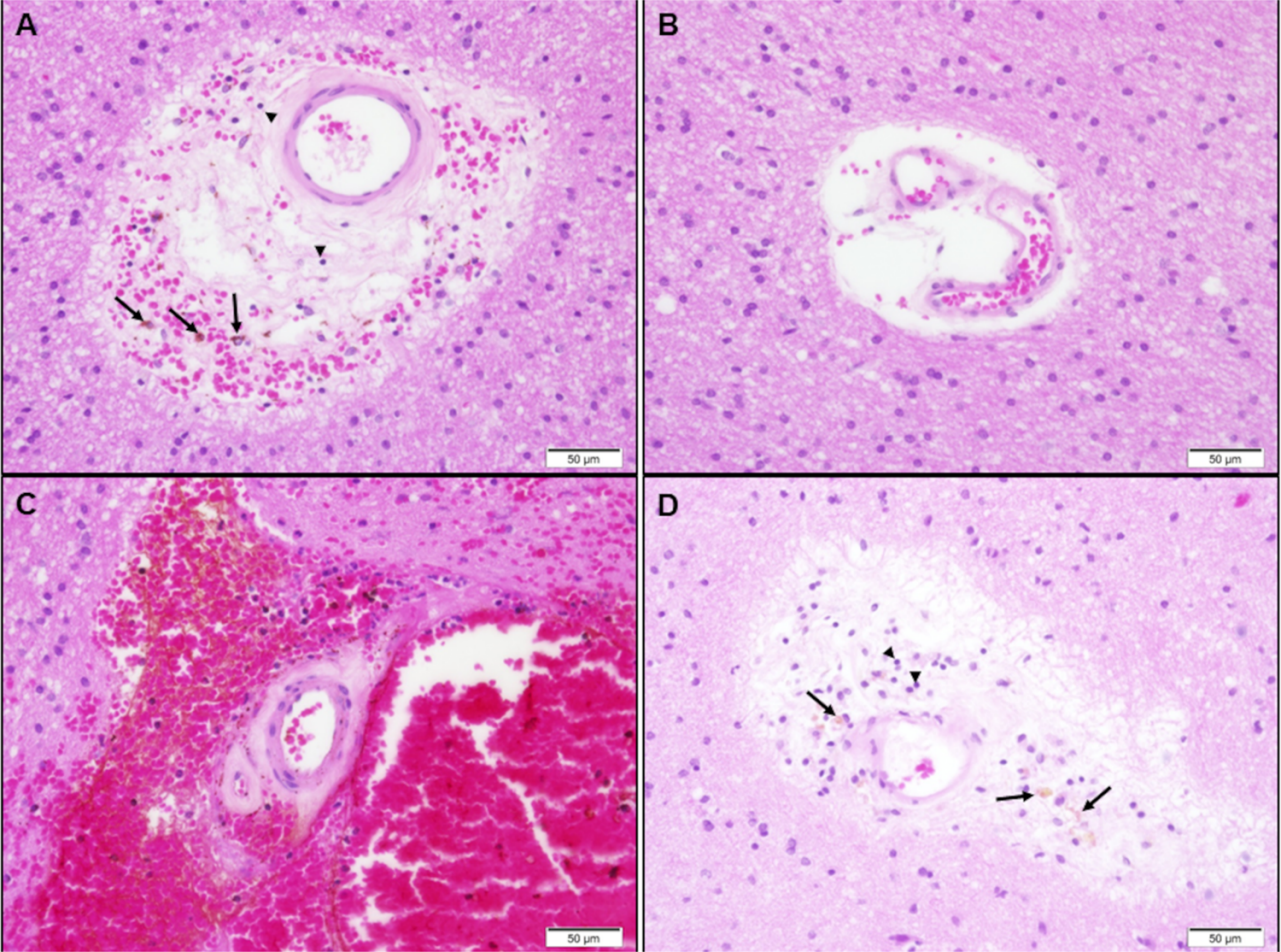
Pathological examination of arterioles within resected epileptogenic tissue from patient 1. Hematoxylin- and eosin-stained, paraffin-embedded sections display white matter blood vessels, with some degree of wall thickening/hyalinization (most prominent in A and C), associated with variable perivascular neuropil loosening (most prominent in B and D), hemorrhage (C), and few hemosiderophages (arrows) and few lymphocytes (arrowheads). Magnification 40×; the scale bar is shown in the lower right corner of each image.

Based on the correlations in abundance across transporters, we postulated that the variability in transporter expression is related to variation in vasculature content and characteristics. Indeed, low abundance of all quantifiable transporters was associated with vascular malformations or arteriolosclerosis-like changes in white matter, which may represent the minimal hypoxic-ischemic type of injury secondary to prolonged epilepsy: calcified vascular malformation (patient 4; abundance of all transporters below LLOQ), cavernous hemangioma (patient 6; all drug transporters below LLOQ), and meningioangiomatosis (patient 2; only SLC29A1/ENT1 quantifiable). Transporter abundance was highest in the neocortex of patient 3, the only sample reported as morphologically normal, without vascular changes or gliosis. In all other samples, presenting intermediate transporter abundance, either severe (hippocampal sclerosis, remote ischemic changes) or mild (hyaline thickening of blood vessels, gliosis, and loosening of the neuropil; Figure 3) abnormalities were reported. Immunohistochemical analysis demonstrated GLUT1 expression on blood vessels from both the neocortex of patient 1 and tissue adjacent to a brain tumor (control), but the GLUT1 staining was apparently lower in the epileptogenic tissue (Figure S4). More precise quantification would require a large number of samples and controls from healthy brains and electron microscopy.

## Discussion

The current study provided, for the first time, absolute quantitative data of drug transporter protein abundance in whole-tissue homogenates from the human epileptogenic brain. Despite the small number of patients and the limited number of quantifiable transporters, our questions were addressed: first, the total abundance of ABCG2/BCRP exceeded that of ABCB1/MDR1 in all samples, and the ABCB1/ABCG2 ratio was not higher than in healthy human brains, as would be implied from the overexpression of ABCB1/MDR1 but not ABCG2/BCRP. Second, in each of the four patients who underwent resection of the anterior temporal lobe, amygdala, and hippocampus, both ABCB1/MDR1 and ABCG2/BCRP were less abundant in the focus of the epileptogenic zone than in the adjacent, less epileptogenic tissue. Abundance of ABCB1/MDR1, ABCG2/BCRP, and SLC2A1/GLUT1 strongly covaried, suggesting variation in the content of functional vasculature within the tissue samples, further supported by the differences in the histological appearance of blood vessels. Hence, the pathophysiological changes that occur in the epileptogenic brain may involve reduced ability to exchange small molecules between blood and brain tissue, which may contribute to drug resistance and even to the disease pathology. In the following paragraphs, we will discuss our findings with regard to the two questions raised in the “Introduction” section and their significance.

### Is the ABCB1/ABCG2 Abundance Ratio Higher than the Values Previously Observed in the Healthy Human Brain?

A higher ABCB1/ABCG2 abundance ratio would be expected based on the overexpression of ABCB1/MDR1 but not ABCG2/BCRP, as presented in the “Introduction” section. However, in the current study, drug transporter abundance ranked similarly in epileptic and healthy brain tissue, with ABCG2/BCRP expression exceeding that of ABCB1/MDR1 by at least 50%, regardless of whether the sample was obtained from the epileptogenic focus or from adjacent tissue. Thus, even if ABCB1/MDR1 is overexpressed in a subpopulation of blood vessels in epilepsy, it is overall less abundant than ABCG2/BCRP, as was reported previously for microvasculature isolated from healthier brain tissue^12,14^ and from samples taken as far as possible from epileptogenic lesions.^13^ In contrast to ABCB1/MDR1, ABCG2/BCRP proteins form dimers or oligomers, hence the total abundance of ABCG2/BCRP may be lower than that of ABCB1/MDR1.^22^ However, for consistency with the literature, we did not make this correction either. The unaltered ABCG2/ABCB1 ratio compared with the nonepileptic human brain could result from lower sensitivity of the whole-brain homogenate approach for detecting altered abundance of proteins in a cell type, which consists of a small fraction of the tissue mass, or reflects between-vessel heterogeneity in transporter expression, as described below. Theoretically, it may also reflect the overexpression of both proteins, but ABCG2/BCRP has not been shown to be overexpressed in epilepsy.^10,11^ Unfortunately, such comparisons could not be made for MRPs, which were unmeasurable in our samples.

### Are Drug Transporters Differently Abundant in Epileptogenic and Less Epileptogenic Human Tissue?

In each of the patients with mesial temporal sclerosis, the abundance of quantifiable transporters was lower in the hippocampus than in the neocortex. Moreover, the rank changed only modestly for ABCB1/MDR1 after correction for relative SLC2A1/GLUT1 abundance. Thus, even if SLC2A1/GLUT1 was altered in the epileptogenic tissue, the magnitude of change was comparable across samples. These findings disagree with the previously demonstrated ABCB1/MDR1 overexpression in the epileptogenic focus but may be related to baseline between-region differences in transporter abundance. Alternatively, this could reflect the heterogeneous quality of the vasculature within the epileptogenic tissue. This assumption is supported by the lower protein yield in epileptogenic brain tissue as compared to homogenates we prepared from nonepileptogenic brain.^12^ A similar phenomenon was recently demonstrated by our group in brain tissue from patients with Alzheimer’s disease.^31^

### Possible Pathophysiology Underlying Altered Drug Transporter Abundance in Epileptogenic Tissue

The variability in quantifiable drug transporter abundance considerably exceeded the 4–5-fold range calculated in healthy brains.^12^ At least a part of the variability can be attributed to the smaller sample number and to the protein content within tissue samples, which likely reflects pathological alterations. SLC2A1/GLUT1 abundance alone could explain 98 and 97% of ABCG2/BCRP and ABCB1/MDR1 variability, respectively, supporting the microvasculature as the major site of localization of ABCB1/MDR1 and ABCG2/BCRP expression in epilepsy (despite potential overexpression in astrocytes and neurons as well^7,23^). ABCB1/MDR1 has been previously colocalized with SLC2A1/GLUT1 in normal rat brain,^24^ postmortem sections of the human brain,^25^ and capillaries freshly isolated from the brains of patients with drug-resistant temporal lobe epilepsy.^26^ Bcrp was localized with both Mdr1 and Glut1 in the mouse brain.^27,28^ An alternative explanation would be coregulation of SLC2A1/GLUT1, ABCB1/MDR1, and ABCG2/BCRP expression, but this is less likely given the different transcriptional regulatory pathways of these transporters.^29,30^ In comparison, the correlation with the nucleoside transporter SLC29A1/ENT1 was not as tight as that with ABCB1/MDR1 and ABCG2/BCRP because SLC29A1/ENT1 is present in cell types other than endothelial cells.^12^

Previously, Cornford and colleagues identified two configurations of brain capillaries with high and low numbers of glucose transporter epitopes in samples from patients with complex partial seizures.^32^ About 25% of the capillary profiles were smaller and exhibited a 10-fold lower number of SLC2A1/GLUT1 proteins.^32^ These findings suggested the existence of variable glucose supply across adjacent small microvolumes of temporal lobe parenchyma, within a distance of micrometers.^32,33^ That finding was later supported by the demonstration of both angiogenesis^34^ and vascular degeneration^35^ in epileptogenic tissue from patients with temporal lobe epilepsy, regardless of the disease etiology. Such heterogeneity in the vasculature might have been overlooked in previous studies that utilized immunohistochemistry with subjective semiquantitative image analysis^5,7,23,36,37^ or computerized analysis based on the application of fixed thresholds.^3,10,38^ Unfortunately, we could not analyze transporter expression in our samples similarly to Cornford et al.^32^ due to the paucity of tissue. Our only indicator of vascular condition was the gross vascular pathologies observed by histological analysis in the tissues with the lowest transporter abundance values and the representative GLUT1 staining. However, our findings imply that *in vitro*-based predictions of drug distribution across the BBB in epileptogenic brain zones should consider potentially impaired vasculature abundance and/or function.

### Revised Model of Small-Molecule Exchange between the Blood and the Epileptogenic Brain

Based on our findings and previous literature,^32,39^ we propose that the distribution of small molecules between the blood and the brain in epilepsy may be highly variable across and within pathologies and patients. Major drug and nutrient transporters may present in certain microvessels within the epileptogenic tissue (e.g., those that highly express SLC2A1/GLUT1) but not in others. Implications of this model might apply to therapeutic choices and to drug discovery: First, in patients with extremely low transporter expression, drugs and drug delivery systems whose penetration across the BBB depends on uptake transporters may not be good candidates for treating epilepsy. In line with this assumption, the monocarboxylate transporter 1, a key transporter for pyruvate, lactate, ketone bodies, and valproate, was deficient in the human epileptogenic hippocampus.^39^ Second, most ASMs are assumed to freely diffuse between the blood and the brain and their cerebral kinetics would not be affected by altered transporter abundance. However, if the area of functional microvessels is reduced, the rate of diffusion may be attenuated as well. This may support nonpharmacological treatment as early intervention, as has been empirically implicated by longitudinal clinical data and the definition of drug resistance following the failure of only two treatment regimens.^1^ Third, transporter depletion might impair the removal of harmful compounds from the brain thereby potentially promoting comorbid conditions. For example, ABCB1/MDR1 contributes to the clearance of cerebral β-amyloid.^40^ Finally, future ASM development may focus on enhancing the binding avidity at target sites, extending the duration of pharmacodynamic effects, and restoring impaired vasculature function.

### Imaging-Based Prediction of Transporter Abundance

Disappointingly, SLC2A1/GLUT1 abundance did not correlate with [^18^F]-activity, possibly because glucose delivery may not be the rate-determining step for its utilization by epileptogenic tissue.^20,21,41^ In addition, low spatial resolution and spill-in within volumes of cubic millimeters might not capture potentially altered function of aberrant blood vessels that characterize chronic, drug-resistant epilepsy. Hence, PET image inspection alone cannot be used as an estimate of tissue SLC2A1/GLUT1 abundance (and related abundance of other BBB transporters) in epileptogenic brain tissue. However, [^11^C]verapamil imaging may emerge as an indicator of low epileptogenic tissue capacity for small-molecule exchange between the blood and the brain, given the fact that our suggested model for small-molecule exchange in epileptogenic brain tissue provides an alternative explanation for the findings obtained by PET imaging of ABCB1/MDR1 activity in patients with epilepsy:^38,42^ based on the transporter hypothesis, reduced distribution of the ABCB1/MDR1 substrate *R*-[^11^C]verapamil into epileptogenic brain tissue has been attributed to increased localized ABCB1/MDR1 activity at the BBB resulting in a higher fraction of the drug effluxed by the BBB. We suggest that the same phenomenon can be additionally explained by reduced delivery of *R*-[^11^C]verapamil and other small molecules to epileptogenic brain regions across impaired vasculature. The latter explanation is also consistent with the reduced impact of ABCB1/MDR1 inhibition on brain *R*-[^11^C]verapamil radioactivity in epileptogenic tissue. The change in *R*-[^11^C]verapamil distribution has been correlated with ABCB1/MDR1 expression,^38^ but only five samples were analyzed. In addition, the use of thresholds for the immunohistochemical analyses could have impaired the ability to detect heterogeneity in transporter expression, as described above.

### Study Limitations

Despite the novelty of quantitative proteomic transporter analysis in human epileptic tissue combined with the individual clinical data, this was a pilot study with a small cohort, with no control nonepileptogenic brain samples. Etiologies and locations of resected brain tissue were variable and perioperative issues, such as withdrawal of ASMs and stress, could have affected transporter expression. However, these represent the real-world population of patients undergoing resective neurosurgeries for epilepsy.

The same lab and personnel previously conducted a targeted proteomic analysis study of BBB transporters in samples from healthy patients. We did not utilize these data as historical controls due to methodological differences between the studies. Particularly, the abundance of drug transporters in homogenates of epileptogenic whole-brain tissue cannot be directly compared to that obtained from microvessel-enriched fractions in the healthy brain.^12,16^ The absence of healthy controls was partially overcome by the comparisons that we performed between patients and between samples from the same patient, and the association with the pathological findings.

Quantification of transporter abundance in tissue homogenates does not provide information as to their localization with regard to cell type, directionality (e.g., luminal vs abluminal), or tissue substructure, including cytoplasmic localization that does not contribute to efflux transport. Yet, homogenates are advantageous in that the loss of material during sample preparation is minimal, hence less error is introduced.^16^ Another strength of the whole-tissue homogenate approach resides in its ability to detect composite changes in transporter abundance that reflects both their BBB abundance and the total fraction of functional microvasculature within the sampled tissue. Our approach, which does not rely on analyses of isolated microvessels, can point, for the first time, to the variation in the content of transporter-expressing vessels in epileptogenic tissue between and within patients. A follow-up study will address the issues of transporter colocalization and identify the exact distribution of transporters within cells and in the tissue, by immunohistochemistry.

An additional limitation is the absence of pharmacogenetic information, especially for *SLC2A1/GLUT1*. Some genetic variants, e.g., common variants of *ABCB1/MDR1* do not affect protein abundance as measured by proteomic analysis,^43^ whereas those in others, e.g., *ABCG2*/*BCRP*, may translate into lower protein levels.^44^ In future studies, we will combine measurements of transporter abundance with their genotyping.

## Conclusions

In the epileptogenic tissue evaluated in this pilot study, SLC2A1/GLUT1, ABCG2/BCRP, and ABCB1/MDR1 abundances may mark the density of functional microvascular endothelium within brain tissue. The epileptogenic tissue might be depleted of transport capacity for drugs and essential compounds, at least in a subpopulation of patients with drug-resistant epilepsy. Impaired drug delivery could be considered when selecting treatments for these patients.

## Supporting Information Available

Transporter protein abundance in epileptogenic brain tissue (the neocortex of patient 3 excluded) (Figure S1); protein–protein correlation of transporter abundance (pmol/g tissue) in epileptogenic brain tissue (the neocortex of patient 3 excluded) (Figure S2); absolute and SLC2A1/GLUT1-normalized transporter protein abundance (Figure S3); and representative GLUT1 staining of paraffin-embedded sections (Figure S4) (PDF)

## Abbreviations Used

ABC: adenosine triphosphate binding cassette
AHL: amygdalohippocampectomy
ASM: antiseizure medication
BBB: blood–brain barrier
BCA: bicinchoninic acid
BCRP: breast cancer resistance protein
CT: computed tomography
DMEM: Dulbecco’s modified Eagle medium
ENT: equilibrative nucleoside transporter
FDG: fluorodeoxyglucose
GLUT: glucose transporter
ILAE: International League Against Epilepsy
LC–MS/MS: liquid-chromatography-tandem mass spectrometry
LLOQ: lower limit of quantification
MDR: multidrug resistance
MRP: multidrug resistance-associated protein
OATP: organic anion transporting polypeptide
*P*-gp: *P*-glycoprotein
PBS: phosphate-buffered saline
PET: positron emission tomography
SLC: solute carrier
VOI: volume of interest
