# Supplementary material for: Abundance of P-glycoprotein and Breast Cancer Resistance Protein Measured by Targeted Proteomics in Human Epileptogenic Brain Tissue
Source: Mol Pharm. 2021 May 19;18(6):2263–73. doi: 10.1021/acs.molpharmaceut.1c00083 (PMC8488956; doi:10.1021/acs.molpharmaceut.1c00083)
Supplement: Supplementary file 1 — mp1c00083_si_001.pdf [file mp1c00083_si_001.pdf]

Abundance of P-glycoprotein and Breast Cancer Resistance Protein Measured by Targeted Proteomics in Human Epileptogenic Brain Tissue

Aniv Mann Brukner, Sarah Billington, Mony Benifla, Tot Bui Nguyen, Hadas Han, Odeya Bennett, Tal Gilboa, Dana Blatch, Yakov Fellig, Olga Volkov, Jashvant D. Unadkat, Dana Ekstein and Sara Eyal.

Supporting Figure 1. Transporter protein abundance in the epileptogenic brain tissue (the neocortex of Patient 3 excluded).

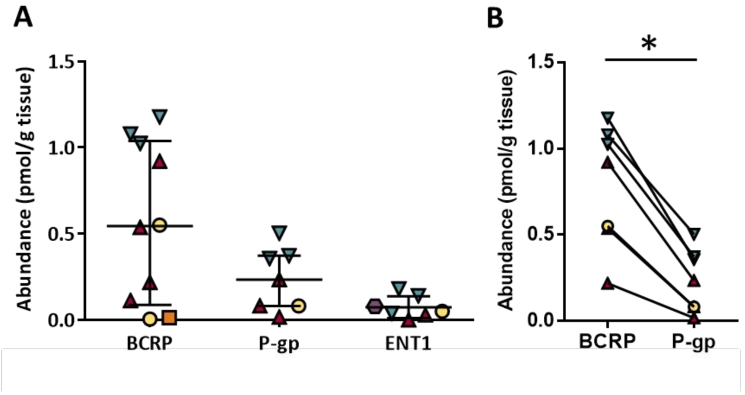

A. Comparative levels of BCRP, P-gp, ENT1 and OATP2B1. Results are presented as median and interquartile range of ten, seven, and seven samples in which BCRP, P-gp, and ENT1 were quantifiable, respectively. **B**. Abundance of BCRP and P-gp in individual tissue samples. * Statistically significant difference, p <0.01, Wilcoxon matched-pairs signed rank test. The representation of samples is as for Figure 1.

Supporting Figure 2. Protein-protein correlation of transporter abundance (pmol/g tissue) in epileptogenic brain tissue (the neocortex of Patient 3 excluded).

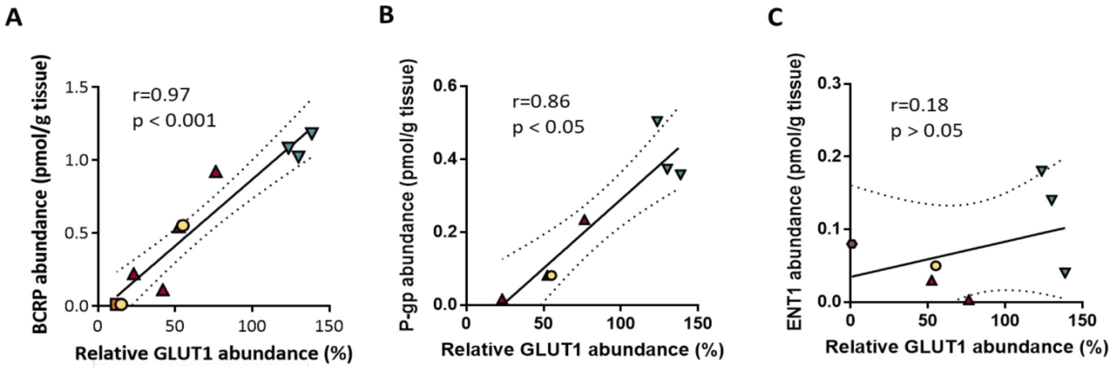

A. GLUT1-BCRP correlation. **B**. GLUT1-P-gp correlation. **C**. GLUT1-ENT1 correlation. Shown are the Spearman r and statistical significance values. The representation of samples is as for Figure 1. The dashed lines represent the 95% confidence band.

Supporting Figure 3. Absolute and GLUT1-normalized transporter protein abundance.

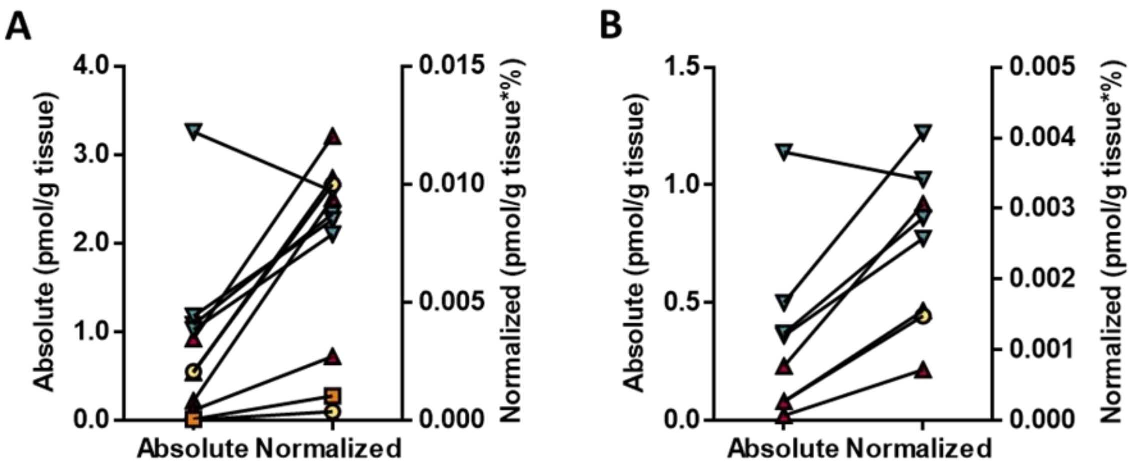

A. BCRP abundance. **B**. P-gp abundance. GLUT1-normalized values are transporter abundance (pmol/g tissue) divided by relative GLUT1 abundance (% of mean GLUT1 values). The representation of samples is as for Figure 1.

Supporting Figure 4. Representative GLUT1 staining of paraffin embedded sections.

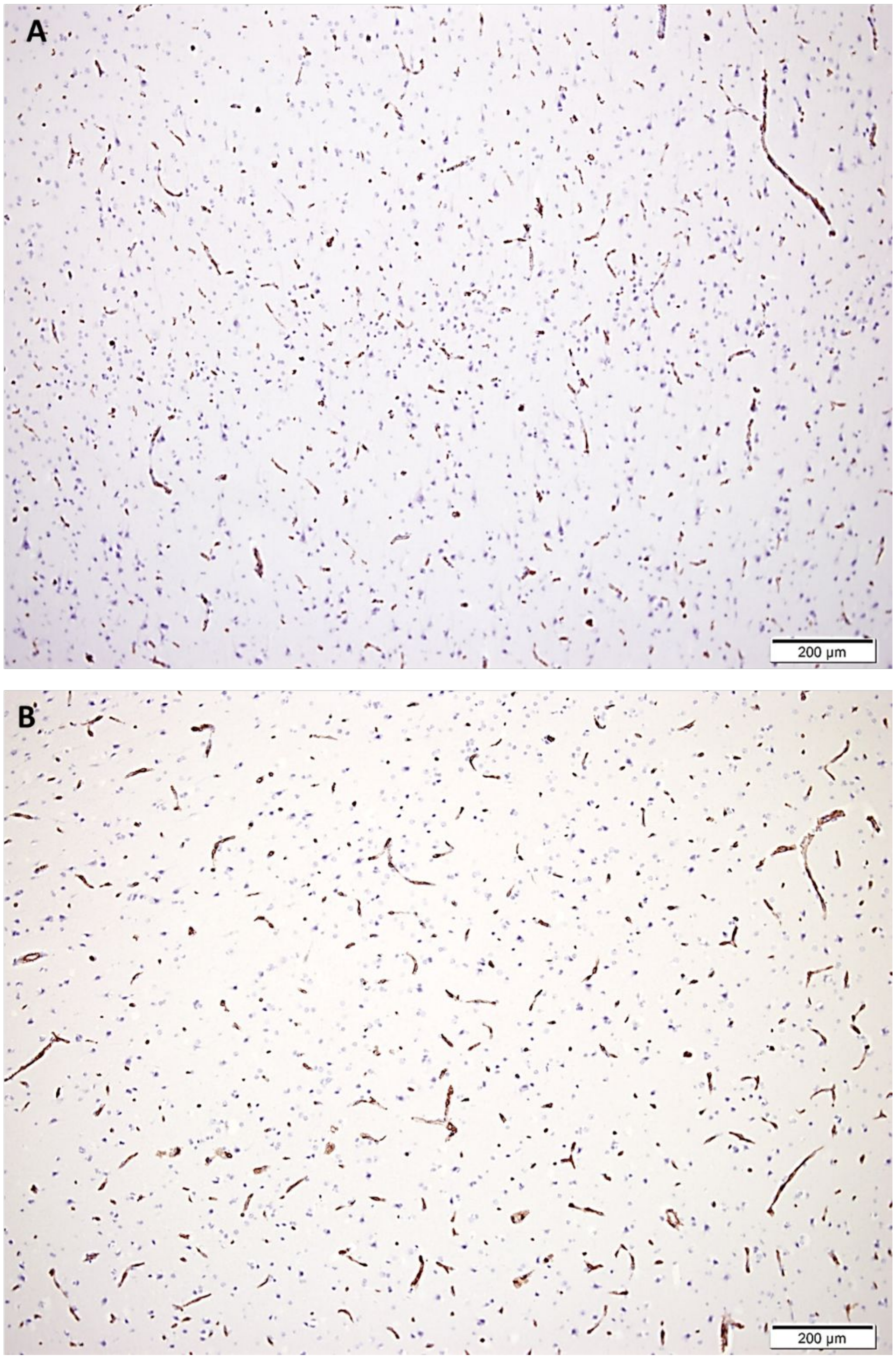

A. Epileptogenic brain tissue from the neocortex of Patient 1 showing overall lower density of GLUT1 (brown) staining. **B**. Normal histologically-appearing brain tissue adjacent to a brain tumor. GLUT1 was stained with polyclonal anti-GLUT1 from Cell Marque, Rocklin, CA, USA; diluted 1:100 and incubated for 40 Min at 37°C.
